# Complementary studies detecting classical bovine spongiform encephalopathy infectivity in jejunum, ileum and ileocaecal junction in incubating cattle

**DOI:** 10.1186/1297-9716-44-123

**Published:** 2013-12-21

**Authors:** Christine Fast, Markus Keller, Anne Balkema-Buschmann, Bob Hills, Martin H Groschup

**Affiliations:** 1 Friedrich-Loeffler-Institut, Institute of Novel and Emerging Infectious Diseases, Südufer 10, 17493 Greifswald-Insel Riems, Germany; 2 Health Canada, Transmissible Spongiform Encephalopathy Secretariat, Suite 14, AL 3000A, 11 Holland Cross, Ottawa, Ontario K1A 0 K9, Canada

## Abstract

Recently we have described the distribution of bovine spongiform encephalopathy (BSE) infectivity and/or PrP^Sc^ in Peyer’s patches (PP) of the small intestine of orally BSE infected cattle. In this follow-up study additional jejunal and ileal PP’s and ileocaecal-junction tissue samples from 1, 4, and 24 months post infection (mpi) were examined by mouse (Tgbov XV) bioassay. Infectivity was demonstrated in ileal PP’s 4 mpi and the distribution/extent of infectivity at 24 mpi was comparable to those seen at earlier time points, revealing no indication for a decline/clearance. These data are relevant for the definition of Specified Risk Materials in the context of the TSE legislation worldwide.

## Introduction, methods, and results

Bovine spongiform encephalopathy (BSE) belongs to the group of transmissible spongiform encephalopathies (TSE) and is associated with the accumulation of a pathological isoform of a host-encoded glycoprotein, prion protein (PrP^Sc^). PrP^Sc^ can be distinguished from its cellular progenitor by its partial proteinase K resistance and hydrophobicity which lead to the formation of fibrils in-vivo and/or in-vitro*.* Classical BSE was transmitted by the oral uptake of infectious feedingstuff [1] to ruminants such as bovines [2] and goats [3], felidae [4] and humans [5]. In the European Union (EU) and in a number of other countries the BSE exposure risk for humans is minimized by the removal of specified risk materials (SRM) from slaughtered cattle that may contain BSE infectivity in incubating animals. Among other tissues this regards, at least in the EU, the intestine. Several authors have described BSE infectivity and/or PrP^Sc^ accumulations in the ileum [6-11], but data on jejunum and colon are patchy [10-12]. In a study we published two years ago [10] we have detected BSE infectivity in jejunum, ileum and ileocaecal-junction of BSE incubating cattle from 8 to 20 months post infection (mpi) with peak levels at 12 mpi. However, immunohistochemistry (IHC) results suggested also the possibility of prion amplification in animals 4 mpi and earlier as well as a time dependent variation in the detectable amounts of PrP^Sc^ up to an age of 24 mpi. This follow up study presented here therefore intended to clarify when BSE infectivity can be found in the small intestine at the earliest time point post infection and also focused on older cattle to prove whether the undulant pattern seen in IHC is reflected by varying infectivity levels. These data are important for a risk-based definition of the gut associated SRM which is needed in the currently ongoing discussion in the EU whether restrictions for the intestinal SRM tissues can be alleviated.

All infection experiments in cattle and mice described in this manuscript were approved by the competent authority of the Federal State of Mecklenburg-Western Pomerania, Germany, on the basis of national and European legislation, namely the EU council directive 86/609/EEC for the protection of animals used for experiments.

As described before [10,13], 56 Simmental calves aged four to six months were orally inoculated with 100 g of a brain stem homogenate pooled from clinically infected BSE cattle. The infectivity load in the homogenate was 10^6.1^ ID_50_/g tissues as determined by end point titration in TgbovXV mice. Eighteen calves inoculated orally with negative brain stem homogenate served as controls. Up to five animals were randomly selected, culled and necropsied after 1, 4, 8, 12 etc. mpi and a wide range of tissue samples was taken under TSE sterile conditions. Specimens from eight infected bovines killed after 1, 4, and 24 mpi and from one control animal (4 mpi) were examined here. Anamnestic data are listed in Table 1. Peyer’s patches from four localizations [10], including two samples from the jejunum and one from each the ileum and ileocaecal-junction were tested for infectivity by mouse bioassay using bovine PrP overexpressing transgenic mice (Tgbov XV) [7]. Fifteen mice per sample were inoculated intracerebrally (30 μL) with 10% tissue homogenates diluted in sterile 0.9% sodium chloride solution. During the incubation time all mice were assessed for the onset of clinical symptoms at least two times per week. Mice showing clinical signs were sacrificed and their brains tested for the accumulation of PrP^Sc^ by a PTA immunoblotting method as described before [10].

**Table 1:** **Anamnestic data (immunohistochemical results are from**[10]**) and mouse bioassay results of all cattle examined**

**Anamnestic data**					**Mouse bioassay (Tgbov XV)**			
**Cow**	**mpi**	**Clinical status**	Results by IHC*		**Jejunum A**	**Jejunum B**	**Ileum**	**Ileocaec-junct**
			**Brain (Obex)**	**Ileum/Ileocaec-junct**				
**KT32**	4	Control	--	−−/−−	0/11	0/15	0/11	0/15
**IT04**	1	Preclinical	--	−−/−−	0/10	0/13	0/13	0/11
**IT44**	1	Preclinical	--	−−/−−	0/13	0/15	0/12	0/13
**IT19**	4	Preclinical	--	+/−−	0/12	0/13	9/15 (380 ± 65)	0/14
**IT45**	4	Preclinical	--	+/−−	0/15	0/14	11/13 (366 ± 46)	0/12
**IT26**	24	Late preclinical	+	++/−−	8/14 (440 ± 96)	0/13	14/14 (317 ± 36)	2/11 (414, 526)
**IT24**	24	Preclinical	--	++/−−	1/13 (533)	0/14	15/15 (328 ± 52)	12/14 (325 ± 45)
**IT47**	24	Preclinical	--	+++/−−	1/15 (448)	2/12 (434, 707)	13/13 (267 ± 20)	3/12 (354,536,725)
**IT58**	24	Preclinical	--	−−/+	0/12	0/15	2/12 (442, 445)	0/15

*All jejunal samples were negative in IHC, *mpi*: months post infection; *IHC*: Immunohistochemistry; -- negative result, +/++ mild/moderate accumulation of PrP^Sc^, Mouse bioassay results relying on PTA-Western blot of mouse brains: the first numbers indicate the number of positive mice/inoculated mice, the number in brackets indicates the mean incubation period in days post infection and the standard deviation.

Results of the PrP^Sc^ analysis and of the bioassay are shown in Table 1. Six out of the eight BSE infected cows carried infectivity at least in one of the gut localizations. The control cow as well as the animals killed one mpi showed no signs of infectivity. The earliest time point of detection was in both cows culled at four mpi. All animals which gave positive results in the mouse bioassay revealed signs of infectivity at least in the ileal sample. Three out of the four cows sacrificed at 24 mpi had accumulated infectivity in jejunal PP’s as well as in the ileocaecal-junction. Animal IT47 (24 mpi) even had accumulated infectivity in all examined intestine samples.

Infectivity levels at the different localizations of the small intestine were unequal, with the highest levels in the ileal PP’s followed by the ileocaecal-junction and jejunal samples. For ileal PP’s (5/6) highest transmission rates (mostly between 66% and 100% of the mice affected) and shortest incubation periods (mean value 327 days, ± 56) were observed in the bioassay. Only one animal (IT58, 24 mpi) displayed sparse amounts of infectivity in this sample. More variable were the results obtained for ileocaecal-junction samples. At this location cow IT24 (24 mpi) carried infectivity levels comparable to those in the ileal PP’s, while two other cows (IT26, IT47, both 24 mpi) showed only low infectivity levels at this site, as indicated by low bioassay transmission rates (two or three mice affected having prolonged incubation periods from 354 up to 725 dpi. Bioassay results for jejunal PP’s were quite diverse with singular diseased mice per group and long incubation periods (440 up to 533 dpi) for most animals 24 mpi, and none in the animals that were sacrificed earlier. However, there was also one cow which was in the late preclinical state, IT 26 (24 mpi), where the jejunal PP’s yielded a bioassay transmission rate of more than 50% with an average incubation time of 440 days.

## Discussion

In this follow-up study we have mapped the exact temporal and spatial emergence and distribution of infectivity in the PP’s of the small intestine in orally BSE infected cattle. According to IHC results described previously [10,11], infectivity was first seen four months after the oral challenge. However in IHC only traces of PrP^Sc^ were seen in single follicles of the ileal PP’s at four mpi [10]. This is not reflected by the results presented here, showing already moderate to high amounts of infectivity comparable to levels at later stages of disease [10]. As the detection of infectivity mostly precedes the detection of PrP^Sc^, these results support the theory that the latent period post exposure during which no detectable infection is present might be around two or three months.

Secondly we were interested in the amount and distribution of PrP^Sc^ at 24 mpi, since the extent of this accumulation varied at earlier time points with peaks at 8 and in particular 12 mpi and lows at 16 mpi respectively [10]. However, these earlier studies did also suggest a higher amount in animals at 24 mpi suggesting an undulant pattern of about 12 months. This finding is now supported by the bioassay results, as three out of the four cattle from the 24 mpi group showed levels and distribution of infectivity comparable to the peaks seen at 12 mpi. However it has to be bear in mind that only four animals per time point were investigated here and in earlier studies and that the variations between individuals are very high [10,14]. This is reflected in the present study by variable detection rates in different animals of the 24 mpi group and might explain the differences seen in infectivity levels reported for ileal samples by several authors before [6,9-11,15].

In summary, data presented here clearly showed that infectivity is not detectable in the small intestine of animals up to four months post experimental oral exposure with an extremely high dose. Moreover, the low amounts of infectivity detectable after the peak at 12 mpi as demonstrated previously, does not imply an irreversible clearance of the infectious agent from the gut over time, but is rather a time-dependent individual fluctuation, as a higher infectivity load is seen again at 24 mpi. Hence, the data presented here are important for a risk- based SRM definition.

## Competing interests

The authors declare that they have no competing interests.

## Author’s contributions

This study was designed by CF, BH and MHG, practically realized by CF, MK, ABB and the data were analyzed and the manuscript was written by CF and MHG. All authors read and approved the final manuscript.
